# Latent Tuberculosis Infection Diagnostic and Treatment Cascade among Contacts in Primary Health Care in a City of Sao Paulo State, Brazil: Cross-Sectional Study

**DOI:** 10.1371/journal.pone.0155348

**Published:** 2016-06-10

**Authors:** Anneliese Domingues Wysocki, Tereza Cristina Scatena Villa, Tiemi Arakawa, Maria Eugênia Firmino Brunello, Silvia Helena Figueiredo Vendramini, Aline Aparecida Monroe, Afranio Lineu Kritski

**Affiliations:** 1 College of Nursing, Federal University of Mato Grosso do Sul, Três Lagoas, Mato Grosso do Sul, Brazil; 2 Department of Public Health, School of Nursing of Ribeirao Preto–University of São Paulo, Ribeirao Preto, Sao Paulo, Brazil; 3 Department of Public Health Nursing and Professional Orientation, School of Medicine of Sao Jose do Rio Preto, Sao Jose do Rio Preto, Sao Paulo, Brazil; 4 Tuberculosis Academic Program, Medical School, Federal University of Rio de Janeiro, Rio de Janeiro, Brazil; 5 Brazilian Tuberculosis Research Network (Rede TB), Rio de Janeiro, Brazil; Public Health Agency of Barcelona, SPAIN

## Abstract

**Background:**

Diagnosis and treatment of latent tuberculosis infection (LTBI) is a tool for global TB control, especially in close contacts. But data is scarce in high burden countries, under field conditions, including data on the benefits of LTBI management.

**Objective:**

To analyze the LTBI diagnosis and treatment cascade among contacts in primary health care (PHC) services in São José do Rio Preto—SP, Brazil.

**Methods:**

Cross-sectional design, conducted with contacts of pulmonary TB patients followed in all PHC services. Data was collected from May to September 2014 in the Reporting System for TB cases (TBWEB) and Reporting System for Chemoprophylaxis. Medical records and treatment follow-up forms were reviewed and all the nurses responsible for TB in PHC services were interviewed.

**Results:**

Among 336 contacts included, 267 (79.4%) were screened for TB or LTBI, according to the presence or not of respiratory symptoms. Among those contacts screened, 140 (52.4%) were symptomatic, 9 (3.4%) had TB disease, 106/221 (48%) had positive TST result, meeting the criteria for LTBI treatment, and 64/106 (60.4%) actually started it. Overall, among 267 screened, only 64 (24%) started LTBI treatment. The completion rates of treatment among the contacts who started it, those with positive TST result and those screened were 56.3% (36/64), 16.3% (36/221) and 13.5% (36/267), respectively. Nurses claimed that asymptomatic TB contacts pay no attention to preventive health care and do not seek medical care as they do not have symptoms of the disease. In reviewing the medical records, high proportions of contacts without evaluation, incomplete assessment, incorrect records of contraindication for LTBI treatment, lack of notes regarding the identification and evaluation of contacts were identified.

**Conclusions:**

There is a need for better organization of the surveillance and investigation routine for contacts in PHC, considering the reorganization of the work process and the features of the local health system.

## Introduction

Diagnosis and treatment of latent tuberculosis infection (LTBI) has the potential to provide both individual benefits (by preventing the morbidity and mortality of active disease) and public health benefits (by preventing further transmission from individuals with reactivated contagious disease) [1,2]. For these reasons, the World Health Organization (WHO) considers treatment of LTBI a tool for global TB control, especially in high-risk groups, such as close contacts, individuals infected with the human immunodeficiency virus (HIV), and patients undergoing immunosuppressive therapy [3].

Despite the evidence in multiple randomized trials that LTBI treatment provides individual benefits, in most settings, this has minimal epidemiologic impact at the population level [4,5].

Under field conditions, in most TB programs, it is estimated that over 90% of the potential benefits of LTBI diagnosis and treatment management are lost as a consequence of patient losses and drop-outs, which occur in each step of the therapy (identification, testing, evaluation, prescription, acceptance and completion) [6,7,8,9,10,11,12].

Brazil still figures among the 22 countries with the highest burden of TB worldwide [13]. The Brazilian Ministry of Health (MoH) recommends decentralization of Tuberculosis Control Program (TCP) activities to the Primary Health Care (PHC) services, in an attempt to enhance access to care and provide patient-centered care [14].

Brazilian guidelines have recommended the expanded investigation of LTBI in contacts of active TB cases and the establishment of LTBI treatment [14]. There is scarce data on the effectiveness of such actions focusing on PHC services though [5,16,17,18,19].

The majority of TB cases are concentrated in the Southeast of Brazil, with São Paulo State (SP) being responsible for 20% of the country’s total burden. São José do Rio Preto (SJRP), a large city in the Northwest of SP, is considered a priority city for TB control, despite its high human and social development index (HDI = 0.834 and Gini index = 0.50). Historically, TB control in the city has been carried out centrally by the local TCP staff in reference centers but, since 2007, all TB control measures have been fully decentralized to the PHC services.

In view of the high burden of tuberculosis in Brazil, together with poor surveillance and routine investigation practices for contacts in PHC, this study aimed to analyze the LTBI diagnosis and treatment cascade among contacts carried out in PHC health services in São José do Rio Preto—SP, Brazil.

## Materials and Methods

This operational research with a cross-sectional design was conducted in 26 PHC services from five health districts of São José do Rio Preto, SP, Brazil. The majority of TB control activities in the city (85%) were conducted by 26 PHC teams, with the exception of TBHIV patients follow-up and drug-resistance specific treatments, which are under the responsibility of reference centers.

PHC teams are composed of at least one physician, one nurse, and a variable number of nursing assistants and community health workers (CHW). There are a total of 11 Basic Health Units (BHU) and 15 Family Health Units (FHU) in the city. In general, BHU are more focused on individual health care, while the FHU is organized to address family and community care in a defined area of maximum 12,000 people. The coverage of FHU in 2013 was only 24.9% of the population.

A contact was defined as "(…) person living in the same place with the index case at the time of pulmonary TB (PTB) diagnosis (…)". The contact investigation starts after notification of PTB patients, who are asked about the existence of contacts and are usually responsible for informing them of the need to attend PHC services for clinical assessment and exams.

The MoH recommends a chest X-ray and a tuberculin skin test (TST) for all children up to 10 years and those asymptomatics aged over 10 years old. For those symptomatic and aged over 10 years old, a sputum smear microscopy (SS) is included to investigate active TB disease. After confirming LTBI, the regimen is composed by a total of 180 doses of Isoniazid (5mg/kg until 10mg/kg, with a maximum dose of 300mg/day) taken in a period of minimum six months and maximum nine months.

Eligible contacts for LTBI screening were contacts of confirmed PTB patients evaluated by the 26 PHC services in the city between January 2012 and December 2013. We excluded contacts of PTB transferred to other cities for follow-up and those individuals without age information. Data was collected from May to September 2014. Information related to LTBI diagnosis and treatment cascade was collected in the Sao Paulo State Recording & Reporting System for TB (TBWEB) and R&R System for Chemoprophylaxis. Medical records and treatment follow-up forms of the index cases were also reviewed through a structured form containing data on the local professional who recorded the following information: presence of identification data, notification of attendance, respiratory symptoms, morbidity of contacts, exams requests and results. Randomly, three records per health service were assessed.

In order to collect further information related to the organization of health services for LTBI diagnosis and treatment cascade, all 26 nurses serving as references to the TB programme in PHC were interviewed, after agreeing to participate in the study and providing their written informed consent. They answered a semi-structured questionnaire, adapted from a validated instrument [20] in Brazil to assess services that develop TB control activities.

The researchers guaranteed the confidentiality of personal identification of individuals and data were completely delinked from any personal identifiers before data analysis.

Data was analyzed using Epidata. Contacts were classified according to age (under and over 10 years old) based on the national guidelines for TB control. Patients subject to Sputum Smear microscopy (SS) were considered as respiratory symptomatic and those with positive TST (>5mm) were classified as corresponding to the criteria for LTBI treatment. The LTBI treatment outcome was classified as: “completed”, when the TB contact ingested 180 doses of Isoniazid in a period of at least six months and up to nine months; “dropout”, when LTBI treatment was stopped before completing these 180 doses of drug; “active TB”, when TB contact became a TB case and “collateral effects”.

The chi-square test (or Fisher's exact test when appropriate) was applied to verify the association between the LTBI diagnosis and treatment cascade performance, the PHC services (BHU and FHS) and the age groups, considering a significance level of 5%.

The study received approval from the Ethics Committee of the São José do Rio Preto Medical School (process 518066/2014) and by the Union Ethics Advisory Group (EAG105/1).

## Results

**Fig 1:**
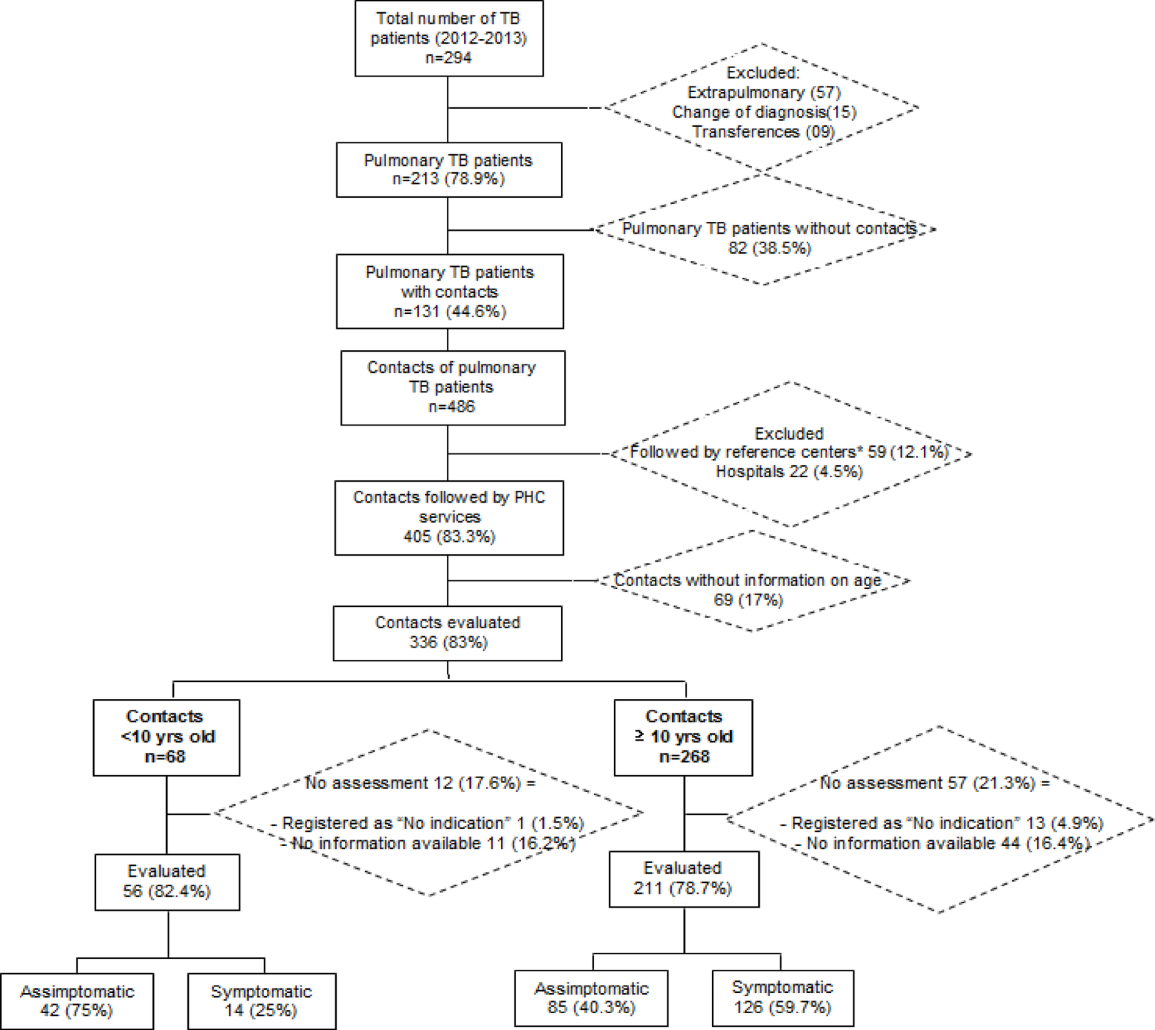
Distribution of notified TB cases and pulmonary TB contacts selected and evaluated, Sao Jose do Rio Preto-SP, Brazil, 2012–2013.

Among 294 TB cases reported, 131 (44.6%) PTB cases with 486 contacts were identified. Among the contacts, 405 (83%) were evaluated at PHC services, being 69 (17%) contacts excluded without age information (Fig 1). Among 336 contacts included, 267 (79.4%) were screened for TB or LTBI, according to the presence or not of respiratory symptoms: 56/68 (96%) of TB contacts up to 10 years of age and 211/267 (78.7%) > = 10 years of age. The majority was female (50.7%) with a median age of 27.6 years [IQ 14–38] (Fig 1). Among the 267 contacts screened, 140 (52.4%) were symptomatic, 9 (3.4%) had TB disease, 106/221 (48%) had positive TST result, meeting the criteria for LTBI treatment, and 64 (60.4%) started it. The completion of treatment among the contacts who started it, those with positive TST result and those screened were 56.3% (36/64), 16.3% (36/221) and 13.5% (36/267), respectively (Table 1).

**Table 1:** Dropouts and losses during follow up in different stages of LTBI diagnosis and treatment Cascade of Care.

							LTBI Treatment				
			Screened*		TST positive		Started	Completed		Completed	Completed
Country (Ref)	Population	Identified (Eligible for screening)	N	% of eligible	N	% of tested & read	N	N	% of started	% of TST positive*	% of screened
Chakaya-Georgia	Contacts	1648^#^	402	24%	212	53%	83	14	18%	2%	3.4%
Rutheford Indonesia	Child contacts	1,585^#^	126	8%	74	59%	82^##^	21	26%	2%	16.7%
Brazil-SJRP Population based	Contacts	336	267	79%	106	48%	64	36	56.3%	16.3%	13.5%

* The number of TST positive is calculated based on: [Rate of positive TST among those tested] times [total eligible population]. If total eligible not known, then % based on reported number who were TST positive.

^#^ In these studies, the number eligible for screening is estimated based on [average number of contacts per case investigated], times [total number of active TB cases known].

The majority of contacts (90.2%) screened were over 10 years of age (79.8%; median age 30.8 years [IQ 13.5–45]) and women (53%) (Fig 1).

**Fig 2:**
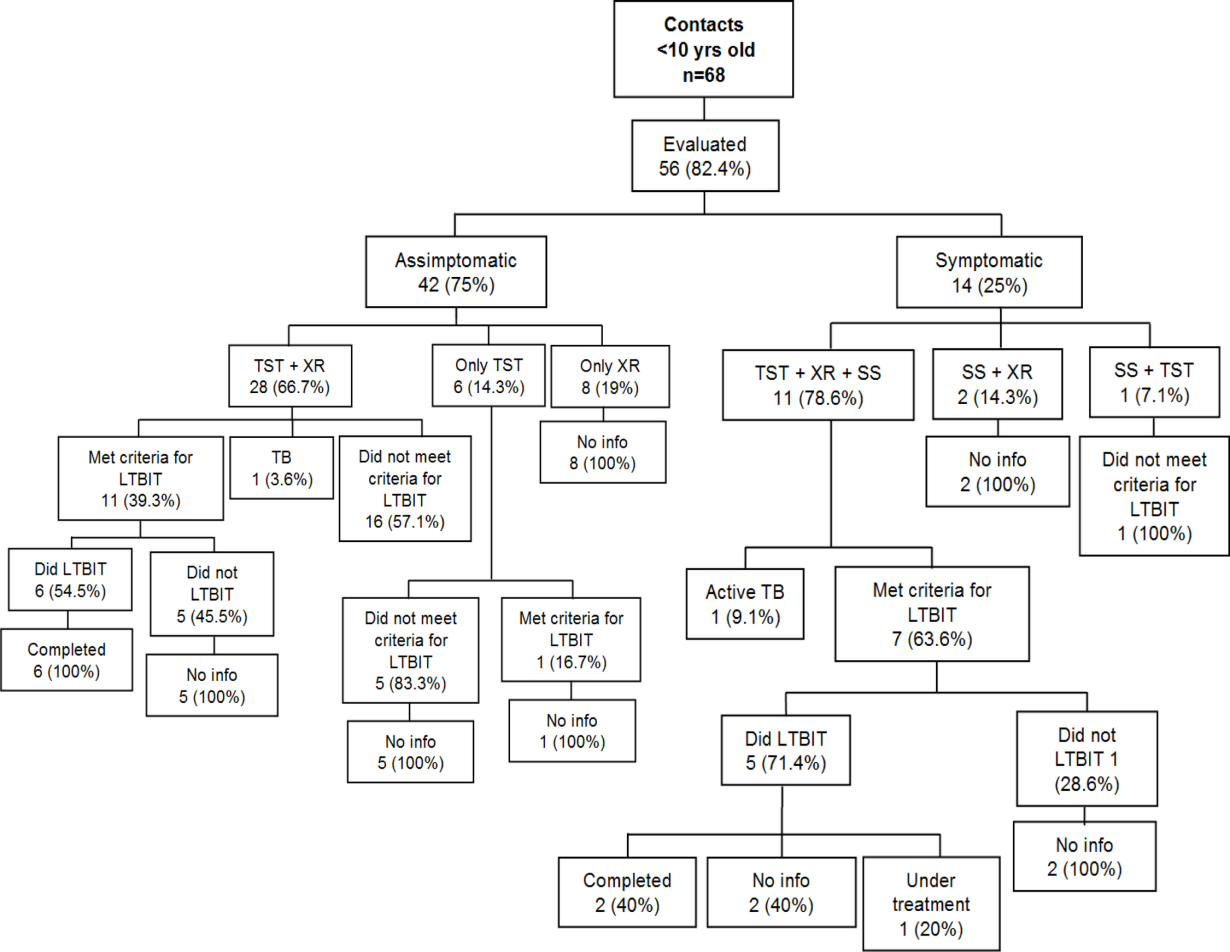
Description of the outcomes of the child contact investigation, Sao Jose do Rio Preto- SP, Brazil, 2012–2013.

Among 56 contacts under 10 years of age, 2 (3.6%) were diagnosed with active TB, 10 (17.8%) were evaluated by smear microscopy and/or chest X ray; 19/46 (41.3%) had positive TST, meeting the criteria for LTBI treatment, but only 11 (57.9%) started the treatment (Fig 2). The completion of treatment among those who started the treatment and among those with positive TST result corresponded to 8/11 (72.7%) and 8/19 (42.1%), respectively.

**Fig 3:**
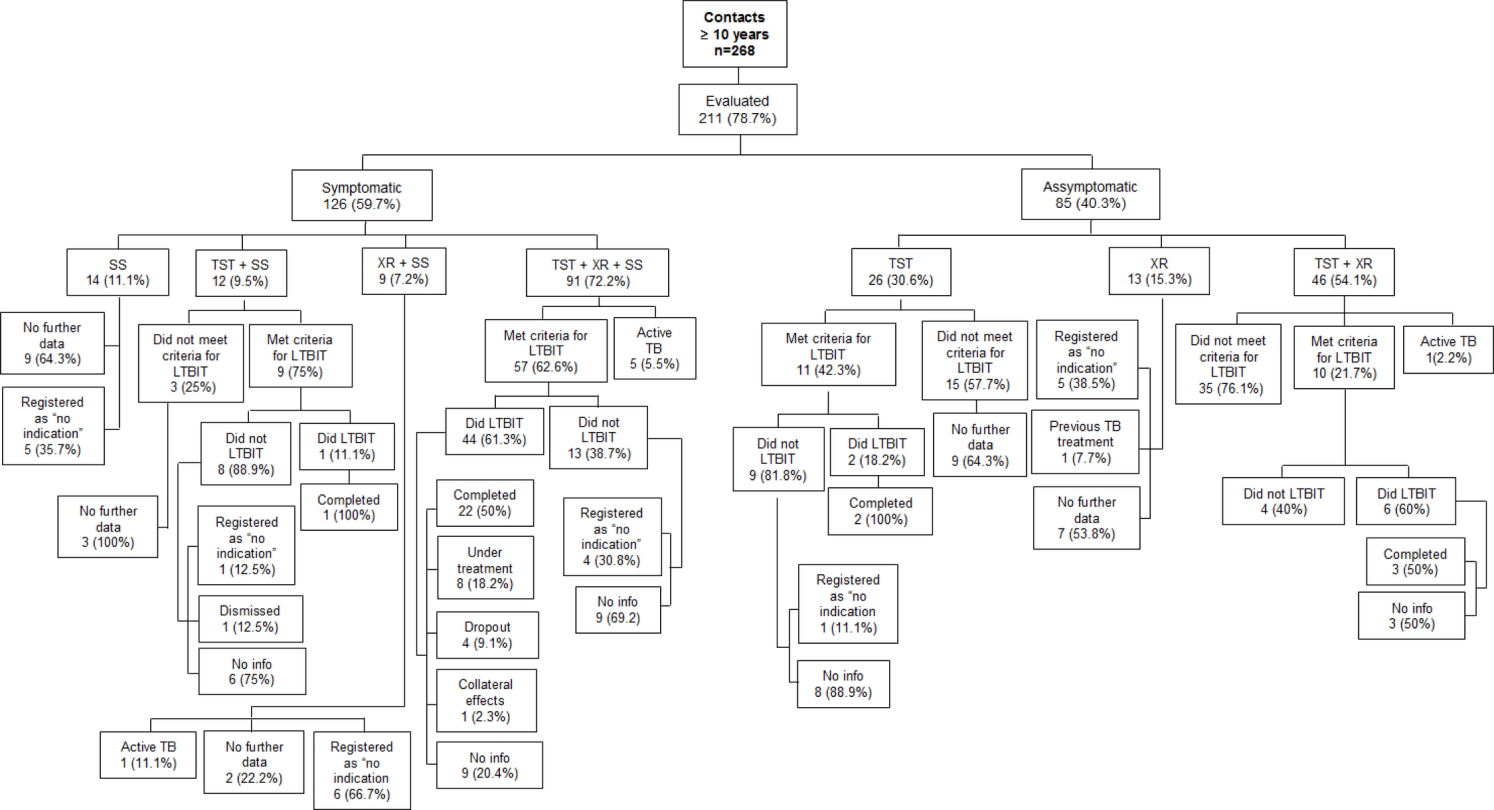
Description of the outcomes of the contact investigation for individuals older than 10 years old, Sao Jose do Rio Preto- SP, Brazil, 2012–2013.

Among 211 contacts over 10 years of age, 7 (3.3%) were diagnosed with active TB, 36 (17%) were evaluated by smear microscopy and/or chest X ray, 87/175 (49.7%) had positive TST, meeting the criteria for LTBI treatment, but only 53 (60.9%) started it (Fig 3). The completion of LTBI treatment among the contacts who started the treatment and among those with positive TST result were, respectively, 28/53 (52.8%) and 28/87 (32.2%). Among the 34 (39.1%) contacts over 10 years of age who did not start LTBI treatment, 6/34 (17.6%) had a record of "no indication to treatment" on the contact evaluation form. The same record was also found among 16 (44.4%) TB contacts not evaluated by TST. Lack of information was another finding throughout the analysis (Figs 2 and 3).

In comparison to BHU, FHS performed more tests to evaluate contacts (only smear microscopy: 99.4% vs 92.2%, p <0.001; just TST: 94.1% vs 86.8%, p = 0.02; chest X ray + TST: 58% vs. 46.7%, p = 0.03), and more frequency of indication to LTBI treatment (36.7% vs 26.3%; p = 0.04), but no difference on the LTBI treatment completion (p>0.05) (Table 2).

**Table 2:** Clinical assessment and exams performed for pulmonary TB contacts according to age and PHC service, Sao Jose do Rio Preto-SP, Brazil, 2012–2013.

		Age			Health service		
Assessment		< 10 yrs old	> 10 yrs old	p	BHU	FHU	p
		(n = 68)	(n = 268)		(n = 167)	(n = 169)	
No exam performed	Yes	12 (17.7%)	57 (21.3%)	0.5090	35 (21%)	34 (20.1%)	0.8489
	No	56 (82.3%)	211 (79.7%)		132 (79%)	135 (79.9%)	
Only SS	Yes	0 (0%)	14 (5.2%)	0.0541^a^	13 (7.8%)	1 (0.6%)	**<0.001**^a^
	No	68 (100%)	254 (94.8%)		154 (92.2%)	**168 (99.4%)**	
Only X-ray	Yes	**8 (11.8%)**	13 (4.8%)	**0.0324**	11 (6.6%)	10 (5.9%)	0.7998
	No	60 (88.2%)	255 (95.2%)		156 (93.4%)	159 (94.1%)	
Only TST	Yes	6 (8.8%)	26 (9.7%)	0.8256	22 (13.2%)	10 (5.9%)	**0.0234**
	No	62 (91.2%)	242 (90.3%)		145 (86.8%)	**159 (94.1%)**	
SS+ X-ray	Yes	2 (2.9%)	9 (3.4%)	0.8629	52 (31.1%)	61 (36.1%)	0.3362
	No	66 (97.1%)	259 (96.6%)		115 (68.9%)	108 (63.9%)	
SS + TST	Yes	1 (1.5%)	12 (4.5%)	0.2508	54 (32.3%)	61 (36.1%)	0.4677
	No	67 (98.5%)	256 (95.5%)		113 (67.7%)	108(63.9%)	
X-ray + TST	Yes	**28 (41.2%)**	46 (17.2%)	**<0.001**	78 (46.7%)	**98 (58%)**	**0.0344**
	No	40 (58.8%)	222 (82.8%)		89 (53.3%)	71 (42%)	
SS + X-ray + TST	Yes	11 (16.2%)	**91 (34.0%)**	**<0.001**	49 (29.3%)	53 (31.4%)	0.6872
	No	57 (83.8%)	177(66.0%)		118 (70.7%)	116 (68.6%)	
Was LTBI treatment indicated?	Yes	19 (27.9%)	87 (32.5%)	0.1932	44 (26.3%)	62(36.7%)	**0.0414**
	No	49 (72.1%)	181 (67.5%)		123 (73.7%)	107(63.3%)	
Was LTBI initiated?**	Yes	11 (57.8%)	53 (60,9%)	0.4781	21 (47.7%)	43 (69.4%)	0.5768
	No	8 (42.1%)	34 (39.1%)		23 (52.7%)	19 (30.6%)	
Was LTBI completed?***	Yes	8 (72.7%)	28 (52.8%)	0.1643	13 (61.9%)	23 (53.5%)	0.9172
	No	3 (27.3%)	25 (47,2%)		8 (38.1%)	20 (46.5%)	

^a^ Fisher’s test

** According to the 106 contacts of pulmonary TB patients to whom LTBI treatment was indicated

*** According to the 64 contacts of pulmonary TB patients who engaged in the LTBI treatment

Interviews were performed with 26 nurses; 22 (84.6%) reported that the contact tracing of PTB patients was a priority in PHC and that this activity was performed for all PTB patients. All of them (100%) mentioned that the information on the index case and contact identification, request for and results of exams, clinical evaluation and notification of attendance were recorded in the follow-up treatment electronic records. Sixteen (61.5%) nurses reported that the recording was also done in the medical records. Sixteen (61.5%) of the nurses included mentioned the challenges to perform the LTBI diagnosis and treatment cascade as follows: workload (64.7%), lack of staff commitment (23.5%), prioritizing index case to the detriment of the contacts (5.9%), and lack of vehicle for home visits (5.9). Additionally, regarding the difficulty to perform exams for contacts at PHC services, 24 (92.3%) nurses claimed that asymptomatic contacts pay no attention to preventive health care and 23 (88.5%) reported that contacts do not seek medical care as they do not have symptoms of the disease.

Among 26 PHC, 72/131 (55%) medical records and forms of PTB were reviewed. Contact tracing information registered by nurses was limited to a list with the name and the tests results (80.6%). There was information regarding health assessment (54.2%), action taken when the contact did not attend the service (58.3%) and reasons for not taking actions in all examinations (54.2%), and no information regarding the presence of respiratory symptoms (100%), BCG vaccination (100%) and co-morbidities (100%). Information regarding the requested exams was found only in the monthly monitoring report sent to the municipal TCP, which is stored in a separate folder in the nurses’ closet.

## Discussion

In our study, high rates of active TB (3.8%), positive TST and indication of LTBI treatment (48.0%) were similar to the rates described in two systematic reviews [21,22] and confirm why contact investigation is strongly recommended for TB control in low and middle-income countries [3]. These data may be underestimated, since there were contacts who were only investigated passively. Additionally, among those screened, only 64 (60.4%) started the LTBI treatment, highlighting an ignored problem. A greater number had already dropped out or were lost during pre-treatment phase, as described in low and middle-income countries. In Georgia and Malawi, among TB contacts screened, only 20.6% and 17%, respectively, started the LTBI treatment [7,10].

The completion of LTBI treatment is as important as the identification of diagnoses and treatment of LTBI among contacts in our study. The number of patients lost during follow-up of LTBI treatment (43.7%) under routine conditions was similar to results reported in another study conducted in Brazil (46.5%) [23], lower than rates in reported in Georgia (82%) [7] and Indonesia (74.4%) [24] and higher than results found in Guinea-Bissau (24%) [25] and Ethiopia (33.7%) [26]. These differences to complete the course of LTBI treatment can be related to individual, socio-cultural and access factors [27].

PHC services are ideal sites for the development of TB control activities because of their attributions of effectiveness (cognitive and technological capacity to address more than 85% of the population problems), communication (ability to order the flow of people, resources and information along the different components of the health system) and accountability (economic and health responsibility for the enrolled population) [28,29,30,31]. In our study, when compared to BHU, LTBI treatment was indicated more frequently among contacts who attended FHS. However, there was no difference in the screening for LTBI and in the treatment completion rates among those contacts screened. It is noteworthy that, although the FHS units have favorable characteristics for an integrated care model, highlighting the family and community health activities performed by the Community Health Worker [15], the work process ended up similar to that seen in the BHU, characterized by weak coordination among healthcare team members’ activities. Additionally, FHS present a high staff turnover and work overload context, providing no increase in TB care access [32], revealing that low organizational aspects and commitment of the health care workers and managers preclude the good performance expected from services. As observed, in our study, the evaluation of other health services based on health records pointed out the difficulties in the use of medical records, usually with low-quality information on the care provided at PHC services [33,34].

Although nurses have reported that TB contact tracing was a priority in PHC, these activities were not carried out. This highlights the prevailing passive nature of PTB contact tracing in both FHU and BHU, consisting of offering information related to the disease and waiting for respiratory symptomatic and PTB contacts to voluntarily seek care and assessment services. Additionally, awareness of the importance of this evaluation is lacking among patients. As a result, contact recruitment is incomplete in most cases. This outcome has also been reported in other similar studies that document screening practices of contacts [35]. Moreover, health education offered to PTB patients is insufficient and inappropriate, which consequently causes delays in TB diagnosis and hinders the identification of LTBI [36].

Active case finding for both active TB and LTBI among contacts has been acknowledged as an important way to identify contacts who could be receiving treatment for LTBI, although this routine has not been incorporated in Brazilian contexts [15,16,21,37,38]. Therefore, the healthcare team has to be prepared to carry out systematic effective health education strategies, as exemplified by intervention trials in Fiji [39] and Nigeria [40], which identified a significant impact in the contact investigation after community interventions [39,40].

In the present study, the low proportion of TB contact tracing activities performed in accordance with Brazilian guidelines and the inconsistency related to LTBI treatment indication in conditions where the TST was not carried out, as well as the absence of information related to the contact tracing, confirm the gaps in the continuity of care provided by PHC, both at BHU and FHS, hampering the identification of those individuals who should receive LTBI treatment.

Data about BCG vaccination was not found in medical records and, although the coverage of BCG vaccination in Brazil is high, data about how long ago the vaccination was performed would be important, in view of the cross-reactivity among TST and BCG. Interferon-gamma release assays (IGRAs) have been considered as an important advance in LTBI diagnosis, especially among BCG-vaccinated individuals, which would be an indication for its widespread use in Brazil. Nevertheless, the high costs make it impossible.

It is known that, to stop TB transmission, early detection is essential. Rapid diagnostic tests, such as GeneXpert MTB / RIF test (Cepheid) have been used in Brazil, but this test has not been implemented yet in the city where the study was undertaken. Considering the high proportion of symptomatic TB contacts found, the implemention of Xpert MTB/RIF should be considered.

Weaknesses in supervision and management of the TCP can also undermine the performance of PHC activities [31]. A strategy that may increase the LTBI diagnosis and treatment cascade is improving the integration among health professionals of the PHC and the TCP, as well as the monitoring of the contact control forms by qualified professionals [30].

Information provided to the PTB patients and contacts has not been used in PHC. No reasons were provided in the medical records on why few contacts did not undergo evaluation as recommended by MoH. This scenario indicates that the work process should be reviewed. Interviews with the PHC nurses identified workload, lack of staff involvement and attention to preventive health by contacts, contacts not feeling sick and physical barriers as constraints to perform TB contact tracing. This finding is similar to another study aimed at assessing the accessibility of tuberculosis treatment and health service performance among TB patients [27]. It is also recognized that PTB contacts have costs and organizational limitations that prevent them from performing all required exams. Therefore, it is useful to make the necessary local arrangements and secure interactions with social and specialized services to provide support, as those factors may be related to low performance of LTBI examinations and treatment [23].

In short, our findings identified errors in the LTBI diagnosis and treatment cascade in PHC, such as the high proportion of contacts with no evaluation, lack of registration and incomplete assessment identified in medical records and forms and incorrect remarks of LTBI contraindication when neither the TST nor any examination had been done. The use of secondary data was considered as a study limitation, since these are subject to weaknesses related to information flow, completeness and validity. Finally, we emphasize the need for a systematic routine for contact tracing carried out at the PHC services, the assessment of the information generated in the PHC, and a higher frequency of monitoring visits by TCP staff to PHC services, providing continuous support for the difficulties those health teams present.

## References

[1] Mandal P, Craxton R, Chalmers JD, Gilhooley S, Laurenson IF, McSparron C, et al Contact tracing in pulmonary and non-pulmonary tuberculosis. Q J Med. 2012;. 105(8): 741–747. 10.1093/qjmed/hcs045 22408150

[2] Tornee S, Kaewkungwal J, Fungladda W, Silachamroon U, Akarasew P, Sunakorn P. Risk factors for tuberculosis infection among household contacts in Bangkok, Thailand. Southeast Asian J Trop Med Public Health. 2004; 35(2): 375–83. 15691140

[3] World Health Organization. 2015. Guidelines on the management of latent Tuberculosis infection Geneva: World Health Organization; 2015. 25973515

[4] Person AK, Pettit AC, Sterling TR. Diagnosis and treatment of latent tuberculosis infection: an update. Curr Respir Care Rep. 2013 12; 2(4):199–207. 25298921 10.1007/s13665-013-0064-y PMC4185413

[5] Rieder HL. Interventions for Tuberculosis Control and Elimination. Paris, International Union against Tuberculosis and Lung Disease, 2002.

[6] Aisu T, Raviglione MC, van Praag E, Eriki P, Narain JP, Barugahare L, et al Ministry of Health. Preventive chemotherapy for HIV-associated tuberculosis in Uganda: an operational assessment at a voluntary counselling and testing centre. U.S National Library of Medicine National Institute of Health. 1995; 9:267–273. 7755915

[7] Chakhaia T, Magee MJ, Kempker RR, Gegia M, Goginashvili L, Nanava U, et al High Utility of Contact Investigation for Latent and Active Tuberculosis Case Detection among the Contacts: A Retrospective Cohort Study in Tbilisis, Georgia, 2010–2011. Plos One. 2014; 9(11):9:e111773 10.1371/journal.pone.0111773 PMC4224404 25379809

[8] Hirsch-Moverman Y, Daftary A, Franks J, Colson PW. Adherence to treatment for latent tuberculosis infection: systematic review of studies in the US and Canada. Int J Tuberc Lung Dis. 2008; 12: 1235–1254. 18926033

[9] Marais BJ, Ayles H, Graham SM, Godfrey-Faussett P. Screening and preventive therapy for tuberculosis. Clin Chest Med. 2009; 30: 827–846. 10.1016/j.ccm.2009.08.012 19925970

[10] Rutherford ME, Ruslami R, Anselmo M, Alisjahbana B, Yulianti N, Sampurno H, et al Management of children exposed to mycobacterium tuberculosis: a public health evaluation in West Java, Indonesia. Bull World Health Organ. 2013; 91:932–941. 10.2471/BLT.13.118414 24347732 PMC3845271

[11] Sharma SK, Mohanan S, Sharma A. Relevance of Latent TB Infection in Areas of High TB Prevalence. Chest. 2012; 142(3):761–73. 10.1378/chest.12-0142 22948580

[12] Van Zyl S, Marais BJ, Hesseling AC, Gie RP, Beyers N, Schaaf HS. Adherence to anti-tuberculosis chemoprophylaxis and treatment in children. Int J Tuberc Lung Dis. 2006; 10(1): 13–18. 16466031

[13] World Health Organization. Global tuberculosis report. Geneva: World Health Organization (WHO), 2012. (WHO/HTM/TB/2012.6). Available: http://apps.who.int/iris/bitstream/10665/75938/1/9789241564502_eng.pdf.

[14] Brasil. Programa Nacional de Controle de Tuberculose. Detectar, tratar e curar: desafios e estratégias brasileiras frente à tuberculose. Boletim Epidemiológico. Secretaria de Vigilância em Saúde- Ministério da Saúde. 2015; 46 (9): 2–19.

[15] Teixeira TP, Mendoza-Sassi RA, Cezar-Vaz MR, Leão LL, Costa SM, Leivas VA. Visita Domiciliar e contatos de pacientes e sua associação com fatores sócio-econômicos e a cobertura pela Estratégia Saúde da Família no município de Rio Grande, RS. Vittalle. 2010; 22(1): 75–85.

[16] Silva DM, Trigueiro DRSG, Medeiros APDS, Oliveira LCS, Nogueira JA. Investigação de comunicantes de tuberculose: desempenho dos serviços de saúde. The FIEP Bulletin. 2012; 82(2): 268–271.

[17] Durovni PBP. Tuberculosis in Rocinha: analysis of epidemiological and operational indicators after 100% coverage of the Family Health Strategy. Master’s Thesis from the Graduate Program in Public Health of the National School of Public Health, Oswaldo Cruz Foundation. 2013. Available: http://bases.bireme.br/cgi-bin/wxislind.exe/iah/online/?IsisScript=iah/iah.xis&src=google&base=LILACS&lang=p&nextAction=lnk&exprSearch=711381&indexSearch=ID.

[18] Caldeira ZMR, Sant’Anna CC, Aide MA. Tuberculosis contact tracing among children and adolescents, Brazil. Rev Saude Púb. 2004; 38(3): 339–345. 10.1590/s0034-89102004000300001 15243661

[19] Mendonça AMC, Kritski AL, Sant'Anna CC. Tuberculosis contact tracing among children and adolescent referred to children's hospital in Rio de Janeiro, Brazil. Braz J Infect Dis. 2015 1 27 19(3):pii: S1413-8670(15)00027-6. 10.1016/j.bjid.2014.12.005 PMC9425340 25636186

[20] Scatena LM, Wysocki AD, Beraldo AA, Magnabosco GT, Brunello MEF, Ruffino-Netto A, et al Validity and reliability of a health care service evaluation instrument for tuberculosis. Rev Saude Publ. 2015; 49:9. 10.1590/S0034-8910.2015049005548 PMC4386554 25741651

[21] Fox GJ, Barry SE, Britton WJ, Marks GB. Contact investigation for tuberculosis: a systematic review and meta-analysis. Eur Resp J. 2013; 41: 140–156. 10.1183/09031936.00070812 PMC3533588 22936710

[22] Morrison J, Pai M, Hopewell PC. Tuberculosis and latent tuberculosis infection in close contacts of people with pulmonary tuberculosis in low-income and middle -income countries: a systematic review and meta-analysis. Lancet Infect Dis. 2008;8(6):359–368. 10.1016/S1473-3099(08)70071-9 18450516

[23] Machado A Jr, Finkmoore B, Emodi K, Takenami I, Barbosa T, Tavares M, et al Risk factors for failure to complete a course of latent tuberculosis infection treatment in Salvador, Brazil. Int J Tuberc Lung Dis. 2009;13: 719–725. 19460247

[24] Rutherford ME, Ruslami R, Maharani W, Yulita I, Lovell S, Crevel RV, et al. Adherence to isoniazid preventive therapy in Indonesian children: A quantitative and qualitative investigation. BMC Res Notes. 2012: 5–7. 10.1186/1756-0500-5-7 PMC3287144 22221424

[25] Gomes VF, Wejse C, Oliveira I, Andersen A, Vieira FJ, Carlos LJ, et al Adherence to isoniazid preventive therapy in children exposed to tuberculosis: a prospective study from Guinea-Bissau. Int J Tuberc Lung Dis. 2011;15: 1637–1643. 10.5588/ijtld.10.0558 22118171

[26] Gebregergs GB, Alemu WG. Household Contact Screening Adherence among Tuberculosis Patients in Northern Ethiopia. PLoS One. 2015; 10(5): e0125767 10.1371/journal.pone.0125767 25955517 PMC4425649

[27] Arakawa T, Arcêncio RA, Scatolin BE, Scatena LM, Ruffino-Netto A, Villa TCS. Accessibility to tuberculosis treatment: assessment of health service performance. Rev Latino-Am. Enfermagem. 2011;19:994–1002. 10.1590/s0104-11692011000400019 21876953

[28] Lima LM, Schwartz E, Cardozo-Gonzáles RI, Harter J, Lima JF. The tuberculosis control program in Pelotas/RS, Brazil: home contact investigations. Rev Gaucha Enferm. 2013; 34(2): 102–110. 24015468 10.1590/s1983-14472013000200013

[29] Mendes EV. O cuidado das condições crônicas na atenção primária à saúde: o imperativo da consolidação da estratégia da saúde da família Brasília: Organização Pan-Americana da Saúde, 2012. 512 p.

[30] Wakiyama TP, Pinheiro AN, Santos AM. Controle dos comunicantes de pacientes com tuberculose pulmonar em uma unidade de tratamento de São Luís (MA) em 2008 e 2009. Rev. Ciênc. Saúde. 2012; 14(1): 15–20.

[31] Hartwig SV, Ignotti E, Oliveira BFA, Pereira HCO, Scatena JH. Evaluation of surveillance of contacts of new tuberculosis cases in the state of Mato Grosso–Brazil. J Bras Pneumol. 2008; 34(5): 298–303. 18545826 10.1590/s1806-37132008000500009

[32] Scatena LM, Villa TCS, Ruffino-Netto A, Kritski AL, Figueiredo TMRM, Vendramini SHF, et al Difficulties in the accessibility to health services for tuberculosis diagnosis in Brazilian municipalities. Rev. Saude Pública. 2009; 43(3): 389–97. 19360234

[33] Vasconcellos MM, Gribel EB, Moraes IHS. Registros em saúde: avaliação da qualidade do prontuário do paciente na atenção básica, Rio de Janeiro, Brasil. Cad. Saúde Pública. 2008; 24: 173–82. 10.1590/s0102-311x2008001300021 18660902

[34] Pereira ATS, Noronha J, Cordeiro H, Dain S, Pereira TR, Cunha FTS, et al O uso do prontuário familiar como indicador de qualidade da atenção nas unidades básicas de saúde. Cad. Saúde Pública. 2008; 24: 123–33. 10.1590/s0102-311x2008001300017 18660897

[35] Thanh THT, Ngoc SD, Viet NN, Van HN, Horby P, Cobelens FG, et al A Household survey on screening practices of household contacts of smear positive tuberculosis patients in Vietnam. BMC Public health. 2014; 14:713 10.1186/1471-2458-14-713 25015682 PMC4226947

[36] Villa TCS, Ponce MAZ, Wysocki AD, Andrade RLP, Arakawa T, Scatolin BE, et al Early diagnosis of tuberculosis in the health services in different regions of Brazil. Revista Latino-Am. Enfermagem. 2013; 21: 190–198. 10.1590/s0104-11692013000700024 23459907

[37] Duarte R, Neto M, Carvalho A, Barros H. Improving tuberculosis contact tracing: the role of evaluations in the home and workplace. Int J Tuberc Lung Dis. 2012; 16 (1): 55–59. 10.5588/ijtld.10.0511 22236846

[38] Silva DM, Nogueira JA, Sá LD, Wysocki AD, Scatena LM, Villa TCS. Performance evaluation of primary care services for the treatment of tuberculosis. Rev. Esc. Enferm. USP. 2014; 48(6): 1044–1053. 10.1590/S0080-623420140000700012 25626504

[39] Delai MY, Gounder S, Tayler-Smith K, Van den Bergh R, Harries AD. Relationship between education and training activities and tuberculosis case detection in Fiji, 2008–2011. Public Health Action. 2012; 2(4): 142–144. 10.5588/pha.12.0064 26392973 PMC4463072

[40] Ekwueme OC, Omotowo BI, Agwuna KK. Strengthening contact tracing capacity of pulmonary tuberculosis patients in Enugu, southeast Nigeria: a targeted and focused health education intervention study. BMC Public Health. 2014; 14: 1175 10.1186/1471-2458-14-1175 25407379 PMC4289165

